# Physiological and microbiome adaptation of coral *Turbinaria peltata* in response to marine heatwaves

**DOI:** 10.1002/ece3.10869

**Published:** 2024-02-05

**Authors:** Xin Zhai, YanPing Zhang, Jie Zhou, Hao Li, Ao Wang, Li Liu

**Affiliations:** ^1^ College of Fisheries Guangdong Ocean University Zhanjiang China; ^2^ Guangdong Laboratory of Southern Ocean Science and Engineering Zhanjiang China

**Keywords:** bacteria, marine heatwaves, physiological adaptation, *Turbinaria peltata*

## Abstract

Against the backdrop of global warming, marine heatwaves are projected to become increasingly intense and frequent. This trend poses a potential threat to the survival of corals and the maintenance of entire coral reef ecosystems. Despite extensive evidence for the resilience of corals to heat stress, their ability to withstand repeated heatwave events has not been determined. In this study, we examined the responses and resilience of *Turbinaria peltata* to repeated exposure to marine heatwaves, with a focus on physiological parameters and symbiotic microorganisms. In the first heatwave, from a physiological perspective, *T. peltata* showed decreases in the Chl *a* content and endosymbiont density and significant increases in GST, caspase‐3, CAT, and SOD levels (*p* < .05), while the effects of repeated exposure on heatwaves were weaker than those of the initial exposure. In terms of bacteria, the abundance of *Leptospira*, with the potential for pathogenicity and intracellular parasitism, increased significantly during the initial exposure. Beneficial bacteria, such as *Achromobacter arsenitoxydans* and *Halomonas desiderata* increased significantly during re‐exposure to the heatwave. Overall, these results indicate that *T. peltata* might adapt to marine heatwaves through physiological regulation and microbial community alterations.

## Graphical abstract

By integrating physiological responses and microbial community analysis, this study reveals that *Turbinaria peltata* can recover from or adapt to the marine heatwaves by undergoing physiological changes and modulating their microbial community.

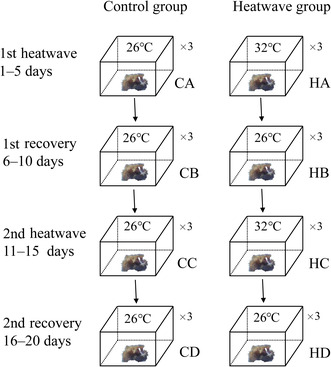

## 1 INTRODUCTION

Marine heatwaves (MHWs) are extreme climate events involving anomalously high surface temperatures, which might last for days to months from local to regional scales (Hughes et al., 2017). With ongoing climate change, MHWs have become more frequent and severe, resulting in escalating damage to coral reefs (Dietzel et al., 2020; Hughes, Anderson, et al., 2018; Hughes, Kerry, et al., 2018; Oliver et al., 2018). These MHWs lead to abnormally high water temperatures, causing corals to bleach by expelling the symbiotic algae within a short period of time, thereby affecting the survival and reproduction of corals and even destroying the entire coral ecosystem (Hoegh‐Guldberg et al., 2007; Hughes, Anderson, et al., 2018; Hughes, Kerry, et al., 2018)

A number of studies have found that corals have some degree of tolerance to high temperatures and may survive under short‐term heat stress (Hughes et al., 2003). Corals respond to high temperatures by various mechanisms. For example, they might adjust their zooxanthellae density or ratio (Barker, 2018; Yu et al., 2020), modify the composition of membrane lipids, particularly by increasing levels of unsaturated fatty acids with double bonds, and enhance the production of antioxidant substances, enzymatic antioxidants such as superoxide dismutase (SOD), catalase (CAT), and glutathione‐*S*‐transferase (GST), as well as non‐enzymatic antioxidants, such as vitamins, carotenoids, and tocopherols (Krueger et al., 2017; Kultz, 2020). These adaptive mechanisms bolster the ability of corals to withstand stress and protect the integrity of their cell membrane structures.

Several studies have highlighted the critical role of symbiotic bacteria in coral survival under heat stress (Claar et al., 2020; Sun et al., 2023). Previous studies have found that alterations in the composition and function of symbiotic bacteria might facilitate the adaptation and ecological plasticity of coral under rapid environmental changes (Frade et al., 2016; Lee et al., 2016; Neave et al., 2016; Roder, Arif, Bayer, et al., 2014; Roder, Arif, Daniels, et al., 2014).

Due to global warming, MHWs are increasing (Frolicher et al., 2018; Oliver, 2019; Oliver et al., 2019). Although numerous studies have evaluated the mechanisms underlying the coral response to MHWs, research on the impacts of repeated exposure to MHWs remains limited (Claar et al., 2020; Marzonie et al., 2023).

*Turbinaria peltata* is a widely distributed reef‐building coral in the Indo‐Pacific region and is an essential and dominant species in the South China Sea. Owing to its resistance to stress, it can be used as a model organism to study the response of the coral holobiont to environmental changes (Xiao et al., 2014). Accordingly, in this study, we investigated the physiological and microbiological effects of repeated MHWs on *T. peltata*. These findings provide valuable data and a reference for understanding the response and mechanisms of adaptation of corals to repeated MHWs.

## 2 MATERIALS AND METHODS

### 2.1 Sample collection

*Turbinaria peltata* specimens were collected from Xuwen Coral Reef National Nature Reserve (109°55′76 E, 20°16′ N). The corals were temporarily housed in a 200‐L tank with a temperature of 26°C, pH of 8.0, and salinity of 33 ppt for 7 days. Artificial seawater was prepared using coral salt from Aquarium Systems (Sarrebourg, France). The corals were illuminated using a full‐spectrum LED lamp (Maxspect, Guangzhou, China) with a light–dark ratio of 12 h:12 h and an effective radiation of 200 μmol (m^−2^ s^−1^). After acclimation, *T. peltata* colonies were fragmented into 48 pieces and placed in another 200‐L tank for an additional week until the corals exhibited normal elongation.

### 2.2 Experimental design

**FIGURE 1:**
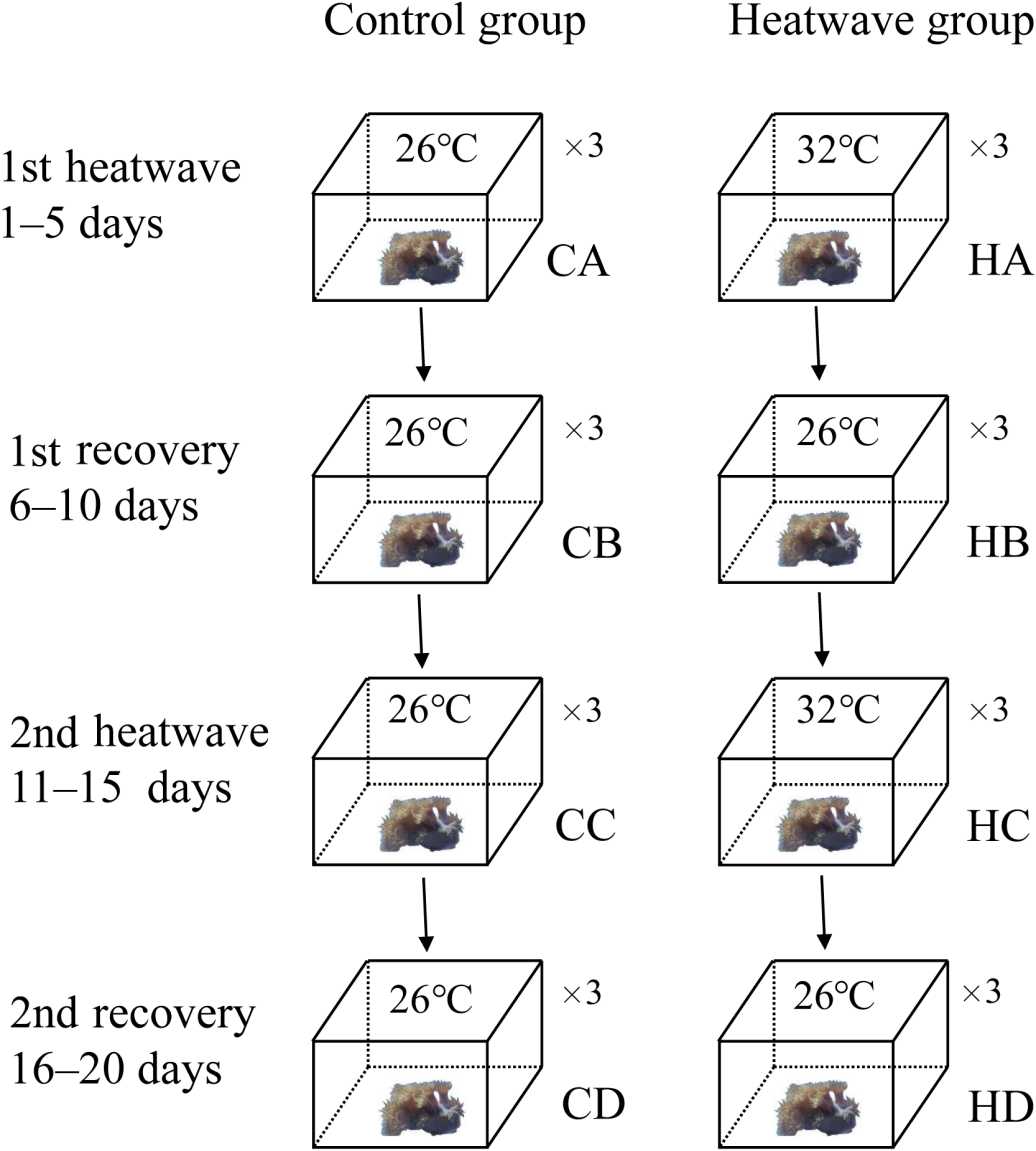
Design of the culture experiment.

A heatwave treatment was conducted by manipulating the seawater temperature over a specific period (Figure 1). Coral nubbins were randomly divided into six aquariums, each with a capacity of 20 L. Two experimental groups were established, a control group (“C”) and heatwave group (“H”), based on historical high‐temperature extremes recorded in Xuwen over the past 10 years (source: http://gd.cma.gov.cn).

From Day 1 to Day 5, a microcomputer digital thermostat (XH‐W1308; Tyrell, Shenzhen, China) was used to gradually increase the seawater temperature from 26 to 32°C, representing the first heatwave. Subsequently, from Day 6 to Day 10, the seawater temperature was returned to 26°C, simulating the first recovery period. From Day 11 to Day 15, the seawater temperature was increased again to 32°C to model a second heatwave. Finally, from Day 16 to Day 20, the seawater temperature was lowered to 26°C to simulate the second recovery period. In the control treatment, the seawater temperature was maintained constantly at 26°C throughout the entire exposure period.

“Day 5” (end of the first heatwave), “Day 10” (end of the first recovery period), “Day 15” (end of the second heatwave), and “Day 20” (end of the second recovery period) were denoted A, B, C, and D, respectively. From each group, three corals were randomly selected for sampling.

### 2.3 Physiological indicators

The coral samples were subjected to a gentle wash using a dental scaler, employing an equal volume of sterile seawater to dislodge the coral tissue from the skeleton. The resulting tissue suspension was then evenly distributed into six centrifuge tubes, with each tube containing 12 mL of the suspension. The centrifuge tubes were then subjected to centrifugation at 4°C and a rotation speed of 4000 rpm for a duration of 10 min. The precipitates were used to determine the density and Chl *a* content of the zooxanthellae, while the supernatants were used to measure the activity of various enzymes. After drying, the surface area of the skeletons was determined using the aluminum foil technique (Fu et al., 2022; Marsh, 1970).

For analyses of the endosymbiont density and Chl *a* content, three portions of the precipitate were washed and suspended in 5 mL of formaldehyde. The endosymbiont density was determined using a microscope with a blood counting plate. The remaining three samples were resuspended in 8 mL of methanol and extracted at 4°C for 24 h. After centrifugation at 4°C for 10 min at 4000 rpm, the Chl *a* content was determined using a UV–visible spectrophotometer (Ritchie, 2006).

Supernatants were collected for further analyses of GST, caspase‐3, SOD, and CAT activities using commercial dilution kits (AKPR013U, AKPR027‐1, AKBL006C, AKEN001U; BOXBIO, Beijing, China). Finally, the protein concentrations were determined using a Bradford kit. Enzyme activity units were normalized to U mgprot^−1^. The data conversion was performed using Excel 2016 Enzyme activity data were presented as the mean ± standard deviation (SD). Graphs were generated using GraphPad Prism 8.0.2. A two‐way analysis of variance (ANOVA) was conducted to assess the differences between the groups.

### 2.4 Microbiological analysis

Genomic DNA was extracted from a 0.5 g frozen coral sample using the TGuide S96 magnetic bead method and the DP812 DNA Extraction Kit (Tiangen Biotech Co., Ltd., Beijing, China). The V3–V4 variable region of the bacterial 16S rRNA gene was PCR‐amplified with the forward primer 27 F (5′‐AGRGTTTGATYNTGGCTCAG‐3′) and the reverse primer 1492 R (5′‐TASGGHTACCTTGTTASGACTT‐3′) (Zhong et al., 2021). Library preparation was performed using the SMRTbell Template Prep Kit (PacBio, Menlo Park, CA, USA), and the PCR was conducted in a Veriti 96‐well thermal cycler (Applied Biosystems, Waltham, MA, USA). Sequencing was carried out on the Sequel II platform after purification.

Raw reads were subjected to filtering to remove adapter sequences using Trimmomatic (version 0.33) and Cutadapt (version 1.9.1) (Bolger et al., 2014; Martin, 2011), yielding clean reads. Paired reads were merged using USEARCH (version 10) (Edgar, 2013), and chimeric sequences were removed using UCHIME (version 8.1). Subsequently, high‐quality sequences were obtained for further analyses. The sequences were clustered at a 97% similarity level using USEARCH (version 10.0) with a default operational taxonomic unit (OTU) filtering threshold of 0.005% of the total read count.

The obtained high‐quality sequences were then annotated against databases, such as Silva, Unite, Greengenes, NCBI, FunGene, and MaarjAM, for taxonomic classification and functional prediction using BugBase and FAPROTAX. Alpha and beta diversity analyses, community composition analyses, and BugBase and FAPROTAX functional prediction analyses were conducted using BMKCloud (www.biocloud.net). Utilizing the QIIME2 software (https://qiime2.org/), we analyzed the Alpha index and validated the significant differences in diversity through a two‐way ANOVA. We also employed the QIIME 1.8.0 software for analyzing to analyze β‐diversity, calculating the distances between samples using the weighted UniFrac algorithm. Based on the matplotlib‐v1.5.1 database, we utilized Python 2 to create species distribution bar charts (at the phylum and species levels). Using the VennDiagram‐v1.6.9 database and R v3.1.1 software, we generated petal venn diagrams. After standardization processing (taking the logarithm) of the OTU data, we selected the top 30 species in terms of abundance and created a heatmap using R. After normalization based on the predicted 16S copy number, we input the Feature table (BIOM format) classified by the Greengenes database was input for BugBase phenotype prediction. Drawing on the current literature on cultivable bacteria, we manually compiled a prokaryotic functional annotation database containing over 7600 functional annotation records from more than 80 functional groups (such as nitrate respiration, methane production, fermentation, and plant pathogens) collected from over 4600 prokaryotic microorganisms. We utilized FAPROTA (version 1.2.6) for a functional prediction analysis with FAPROTAX (Liang et al., 2022). The data that support the findings of this study are openly available in figshare: https://doi.org/10.6084/m9.figshare.24152901.

## 3 RESULTS

### 3.1 Physiological response

**FIGURE 2:**
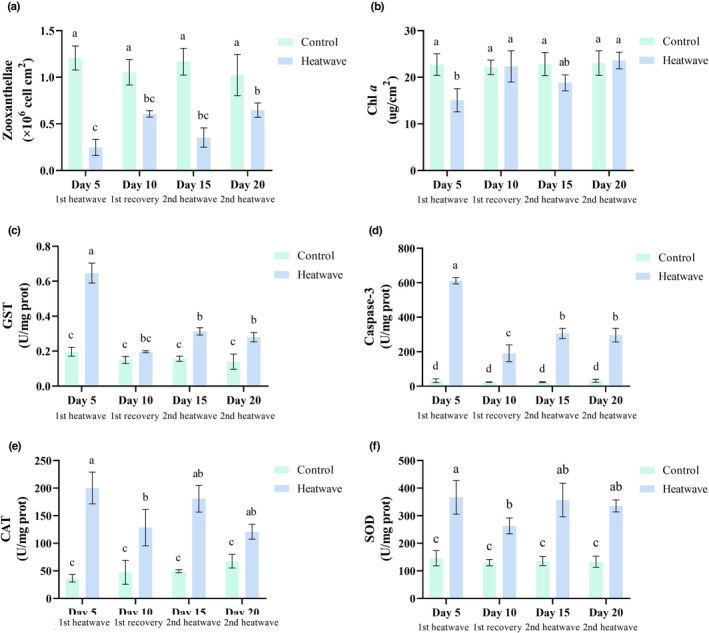
Response of coral physiological indicators under different treatments. (a) Zooxanthellae, (b) Chl a, (c) GST, (d) caspase‐3, (e) CAT, (f) SOD.

*Turbinaria peltata* showed a substantial physiological response to MHW stress (Figure 2). After the first MHW, the density of zooxanthellae was approximately 79.44% lower, and the concentration of Chl *a* was approximately 33.67% lower than those in the control group. The levels of GST, caspase‐3, SOD, and CAT increased significantly (*p* < .05). The effect of the second MHW was less intense than that of the initial MHW, as indicated by the slight decreases in the density of zooxanthellae and concentration of Chl *a* (i.e., approximately 69.71% and 17.63% compared with levels in the control group, respectively). Although levels of GST, caspase‐3, SOD, and CAT increased, the observed changes were not as substantial as those for the first heat stress. Levels of GST and caspase‐3 were significantly lower after the second heat stress than after the first heat stress (*p* < .05). Levels of CAT and SOD were also lower; however, the difference was not significant.

### 3.2 Bacterial diversity analysis

In a high‐throughput sequencing analysis, we obtained 318,431 high‐quality sequences without chimeras. The dilution curve of the *S*_obs_ index reached a plateau, indicating that sequencing data were sufficient and a wide range of microbial species were detected, reflecting microbial diversity.

**FIGURE 3:**
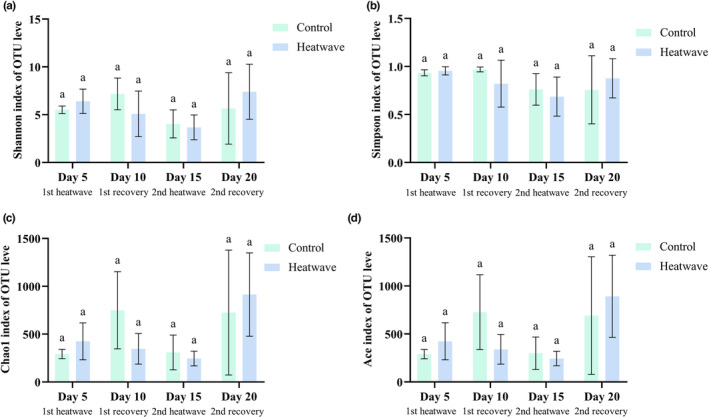
α‐Diversity analysis of coral‐symbiotic bacteria under different treatments. (a) Shannon. (b) Simpson. (c) Chao1. (d) Ace.

At the end of the first heatwave, the Simpson index did not differ from that in the control group. However, the Shannon, Chao1, and Ace indexes were slightly higher than those of the control group (Figure 3). In the second MHW, each index showed a slight decrease compared with levels in the control group.

**FIGURE 4:**
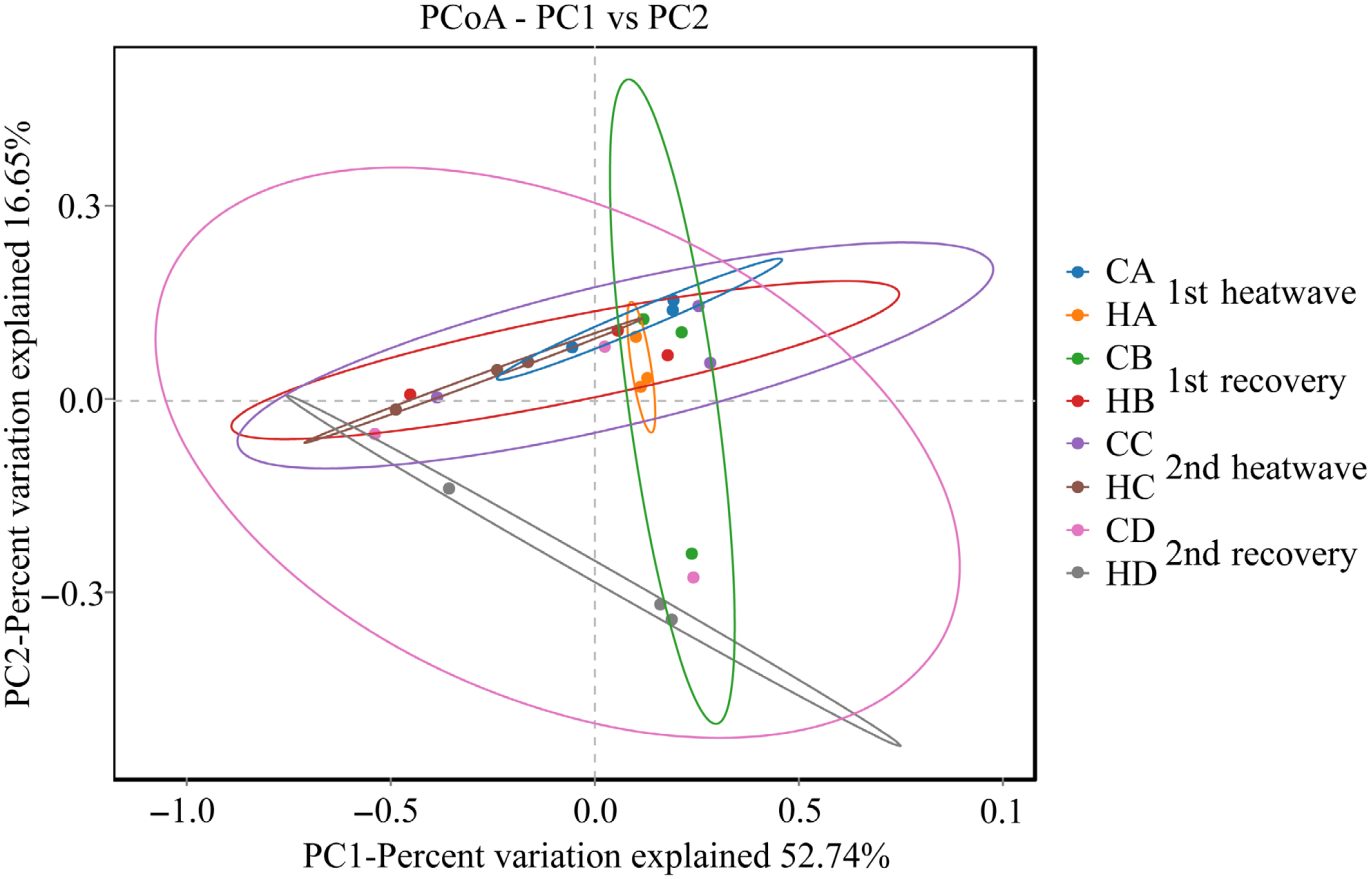
Principal coordinates analysis of coral symbiotic microbial communities under different treatments.

In a principal coordinates analysis (PCoA), the first two axes accounted for approximately 69.39% of the total variance in the microbial community composition among all samples. PC1 explained 52.74% of the variance, while PC2 explained 16.65% (Figure 4). The three replicate samples within each group exhibited close clustering and high repeatability, and more variation was observed between groups than within groups.

### 3.3 Difference in bacterial community composition among groups

**FIGURE 5:**
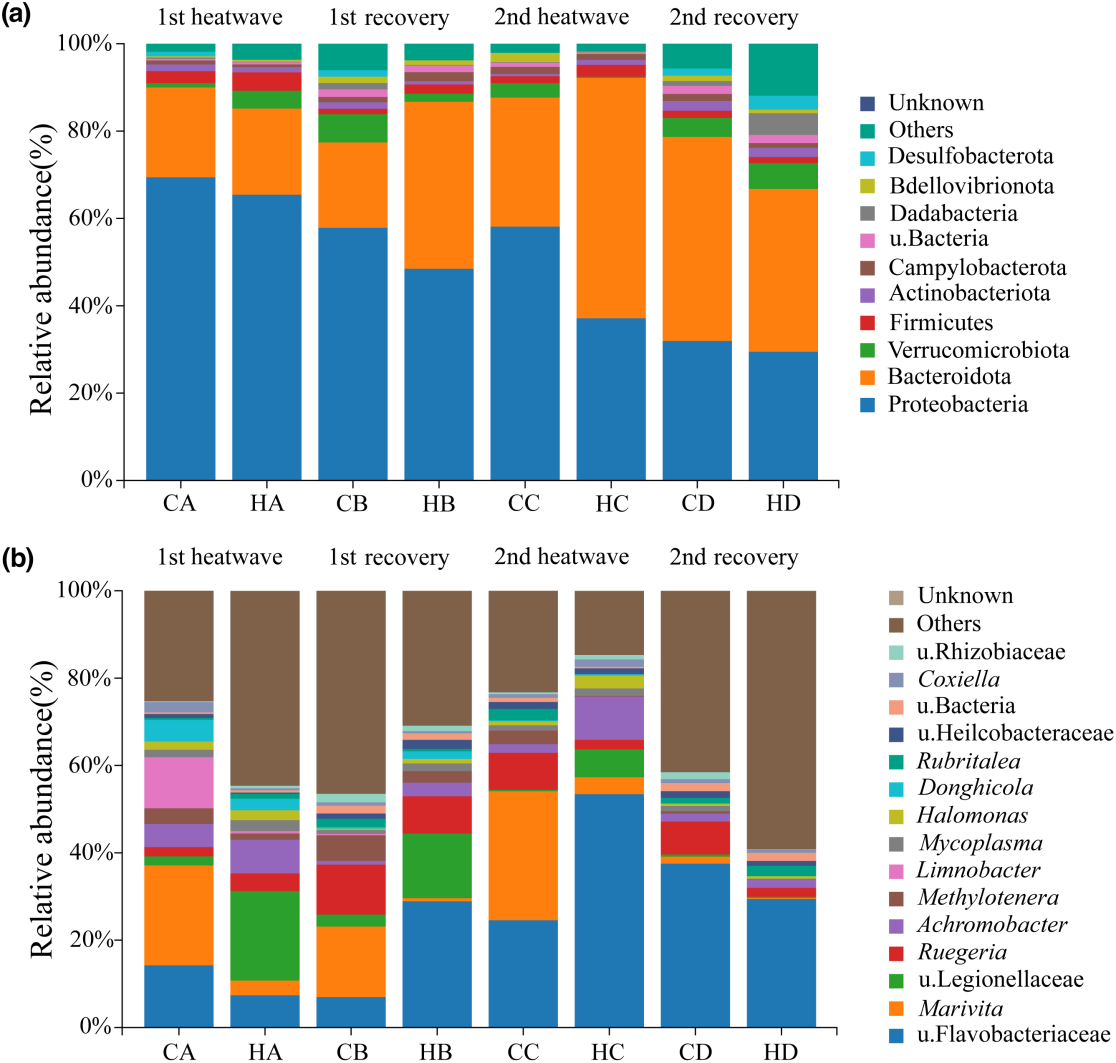
Coral symbiotic bacterial community structure, (a) histogram of the community composition at the phylum level, (b) histogram of the community composition at the genus level. Unclassified species are indicated by u. (unclassified) plus the family name.

An analysis of co‐occurring bacterial communities revealed the presence of 36 phyla, 88 orders, 242 classes, 413 families, 858 genera, and 1422 species across all samples. As shown in Figure 5a, the top 10 bacterial phyla in terms of relative abundance were Proteobacteria, Bacteroidetes, Verrucomicrobia, Firmicutes, Actinobacteria, Campylobacterota, Dadabacteria, Bdellovibrionota, and Desulfobacterota. The remaining 16 bacterial phyla were classified under the “other” category. Proteobacteria and Bacteroidota represent dominant phyla in each group.

We analyzed the distribution of symbiotic microbial communities at the genus level, as depicted in Figure 5b. The top 15 bacterial genera in terms of relative abundance were u. Flavobacteriaceae, *Marivita*, u. Legionellaceae, *Ruegeria*, *Achromobacter*, *Methylotenera*, *Limnobacter*, *Mycoplasma*, *Halomonas*, *Donghicola*, *Rubritalea*, u. Helicobacteraceae, u. Bacteria, *Coxiella*, and u. Rhizobiaceae.

With respect to the relative abundance of coral symbiotic bacteria, after the first MHW, the HA group exhibited increases in u. Legionellaceae, *Ruegeria*, and *Achromobacter* and decreases in u. Flavobacteriaceae, *Marivita*, *Methylotenera*, *Limnobacter*, *Donghicola*, and *Coxiella*. Following the 5‐day recovery period, the HB group showed increases in the relative abundance of u. Flavobacteriaceae, u. Legionellaceae, and *Achromobacter* along with decreases in *Marivita*, *Ruegeria*, *Methylotenera*, and *Rubritalea*. After the second MHW, the HC group displayed increases in the relative abundance of u. Flavobacteriaceae, u. Legionellaceae, *Achromobacter*, and *Halomonas* and decreases in *Marivita*, *Ruegeria*, *Methylotenera*, and *Rubritalea*. Finally, after the second 5‐day recovery, the HD group exhibited an increase in the relative abundance of *Rubritalea* and decreases in u. Flavobacteriaceae, *Marivita*, and *Ruegeria*.

### 3.4 Changes in bacterial phenotypes and functional groups

#### 3.4.1 BugBase forecast

**FIGURE 6:**
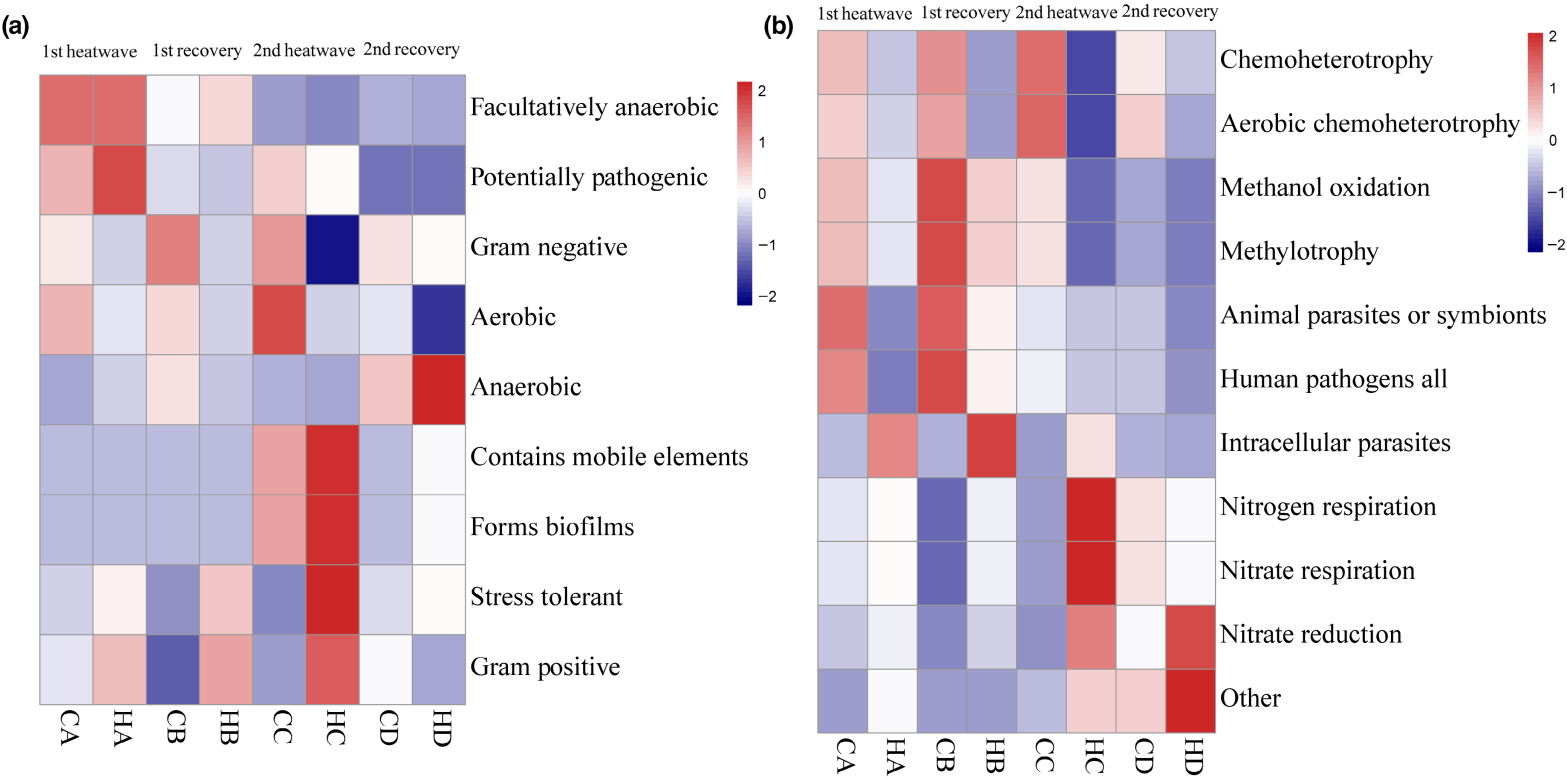
Functional analyses of coral symbiotic microbial communities, (a) BugBase bacterial phenotypic structure analyses, (b) FAPROTAX analyses of the eco‐functional composition.

BugBase can be used to predict the phenotypes of prokaryotic bacteria in environmental samples. As shown in Figure 6a, the predominant phenotypes observed in all samples at the end of the experiment were as follows: gram‐negative, stress‐tolerant, oxygen demand (aerobic, anaerobic, and facultatively anaerobic), gram‐positive, potentially pathogenic, contains mobile elements, and forms biofilms. These results showed that the abundance of microorganisms with different phenotypes differed among treatment periods. Following the initial MHW, there were increases in the relative abundances of potentially pathogenic and gram‐positive bacteria in the HA group. After the 5‐day recovery period, the HB group displayed increases in the relative abundance of stress‐tolerant and gram‐positive bacteria. Subsequently, after the second round of MHW, the HC group showed increases in the relative abundance of taxa associated with the following phenotypes: mobile elements, forms biofilms, stress tolerant, and gram‐positive. Similar patterns were observed in the HD group; however, there was also an elevation in the relative abundance of anaerobic bacteria.

#### 3.4.2 FAPROTAX forecast

We analyzed the ecological functions of prokaryotic bacteria using FAPROTAX. As shown in Figure 6b, the observed symbiotic bacteria displayed a wide range of ecological functions. These functions included intracellular parasites, nitrogen respiration, nitrate respiration, nitrate reduction, and other unidentified functions. These findings provide insights into the functional versatility and contributions of symbiotic bacteria within the ecosystem. The symbiotic microbial functions exhibited distinct changes throughout the experiment. At the end of the initial MHW, the HA group showed a marked increase in the relative abundance of intracellular parasite bacteria. After the 5‐day recovery, the HB group maintained this high abundance of intracellular parasite bacteria. Following the subsequent MHW, the HC group demonstrated a notable increase in the relative abundances of bacteria associated with nitrogen respiration, nitrate respiration, and nitrate reduction. Additionally, after a 5‐day recovery period, the HD group exhibited an elevated relative abundance of nitrate reduction and other bacteria.

### 3.5 Core microbiological analysis

**FIGURE 7:**
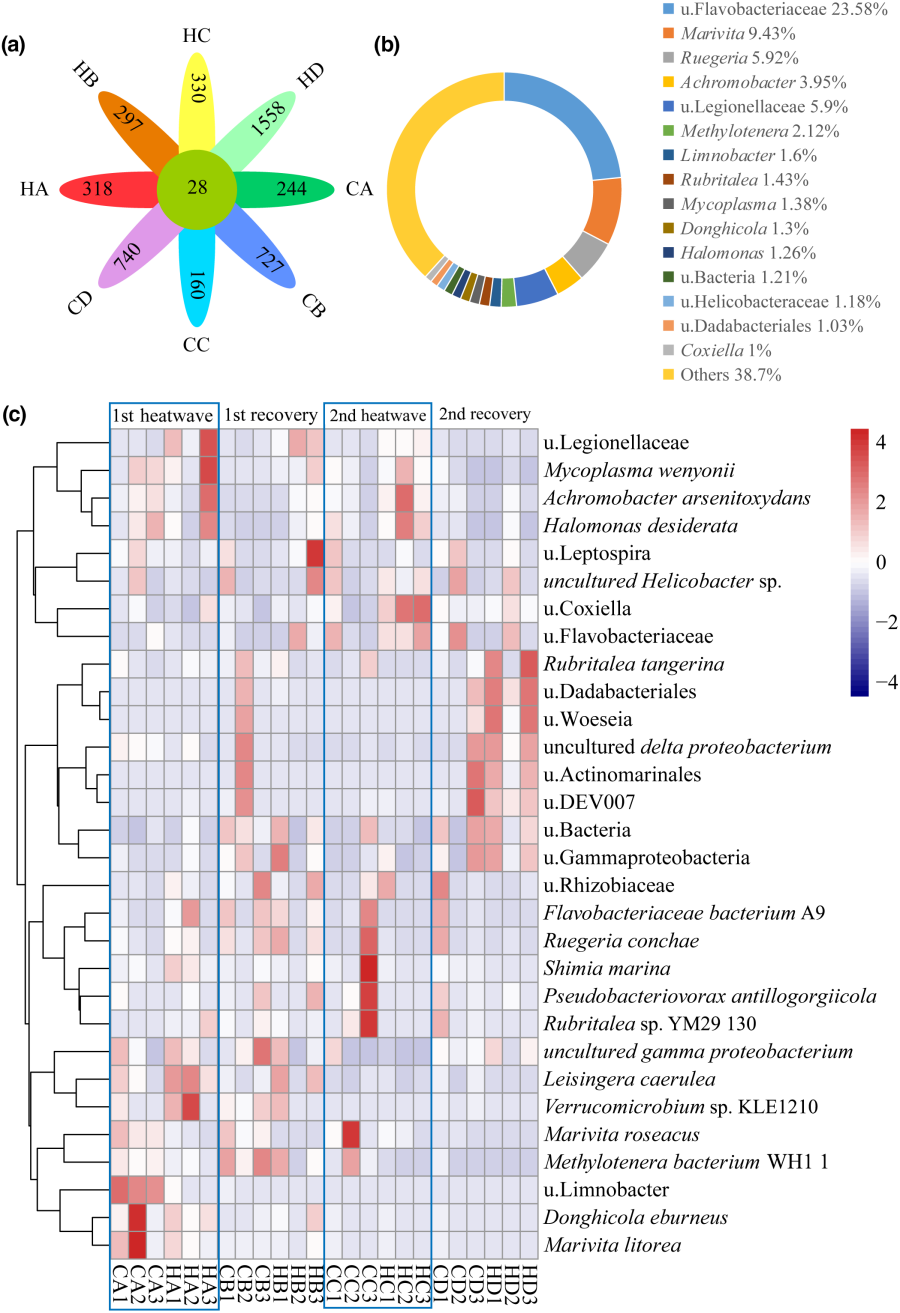
Core bacterial community structure, (a) Venn diagram of differences in community structure at the OTU level. (b) Pie chart of the community composition at the genus level, with bacteria <1% at the genus level classified as others. (c) Heat map of the community composition at the genus level of core bacteria. Taxa not identified to the genus level are indicated by u. (unclassified) plus the family name.

To examine the changes in symbiotic bacteria within corals, 24 samples from the two MHW treatments were analyzed. The distribution of core OTUs was assessed, and 28 core OTUs were identified (Figure 7a). At the genus level, the distribution of these core OTUs, depicted in Figure 7b, was as follows: u. Flavobacteriaceae (23.58%), *Marivita* (9.43%), *Ruegeria* (5.92%), u. Legionellaceae (5.90%), *Achromobacter* (3.95%), u. Methylotenera (2.12%), *Limnobacter* (1.60%), *Rubritalea* (1.43%), *Mycoplasma* (1.38%), *Donghicola* (1.30%), u. Bacteria (1.21%), u. Helicobacteraceae (1.18%), u. Dadabacteriales (1.03%), and *Coxiella* (1.00%), indicating their dominance within the core OTUs.

As shown in Figure 7c, a heat map of the core OTUs at the species level was constructed based on the top 30 species with respect to average abundance. The abundance of *Leisingera caerulea* was higher in the HA group than in other groups, while the HB group displayed increases in u. Leptospira, *Halomonas desiderata*, and u. Flavobacteriaceae. After the second MWH, the HC group exhibited elevated levels of u. Flavobacteriaceae, u. Coxiella, *Halomonas desiderata*, and *Achromobacter arsenitoxydans*. After 5 days of recovery, the HD group demonstrated significant increases in the relative abundances of *Rubritalea tangerina*, u. Dadabacteriales, u. *Woeseia*, uncultured *delta proteobacterium*, u. Actinomarinales, and u. DEV007 bacteria.

These results suggest that MHWs lead to significant changes in coral‐symbiotic microbial communities, and the core microbial communities differ between two heatwaves and between the heatwave and recovery periods.

## 4 DISCUSSION

With the ongoing global warming trend, the frequency of MHWs is expected to increase. Therefore, the ability of corals to adapt to or recover from extreme heat stress will be crucial for their survival. From this study, we found that *T. peltata* adapted to high temperatures, as indicated by a weaker physiological response to the second heatwave than the first heatwave, and the observed changes were accompanied by a decreased abundance of pathogenic bacteria and increased abundance of beneficial bacteria in symbiotic communities.

### 4.1 Physiological adaptation of *Turbinaria peltata* in response to marine heatwaves

Previous studies has shown that some corals are clearly able to adapt to heat stress (Hackerott et al., 2021; Schoepf et al., 2022; Yu et al., 2020). This adaptability primarily relies on the thermal tolerance of their symbiotic algae, with susceptible populations being expelled and remaining populations showed enhanced metabolic activity and photosynthetic efficiency to cope with high temperatures (Arandia‐Gorostidi et al., 2017; Pernice et al., 2012). In our study, the initial MHW resulted in a significant decrease in the density of zooxanthellae (*p* < .05). In our study, initial exposure to MHW resulted in a significant decreases in the zooxanthellae density and Chl *a* content. However, the second MHW had a weaker impact. These findings are consistent with previous research results (Bay & Palumbi, 2015). Furthermore, corals regulate the synthesis and activity of antioxidant enzymes, such as superoxide SOD, CAT, and GST, as well as apoptotic proteases, such as caspase‐3, to counteract acute heat stress (Bhagooli & Hidaka, 2004; Lesser, 2006; Thummasan et al., 2021). Our findings revealed that the initial MHW led to significant increases (*p* < .05) in the levels of GST, caspase‐3, SOD, and CAT. However, the effects were alleviated upon subsequent exposure to MHWs (Yu et al., 2020). This regulatory process helps in clearing excessive reactive oxygen species, thereby maintaining normal cellular function and physiological states. Consequently, repetitive exposure to heatwaves imposes less physiological stress on corals compared to that for the initial exposure (Xu et al., 2021), indicating that *T. peltata* has physiological adaptations to withstand MHWs.

### 4.2 Effects of the initial marine heatwave on bacteria

Symbiotic bacteria play a crucial role in coral reef ecosystems by contributing to coral growth and survival (Ziegler et al., 2017). Our study revealed that Proteobacteria and Bacteroidota were identified as the predominant phyla in each group, which is consistent with previous research findings (Huggett & Apprill, 2019; Rowher et al., 2002). However, environmental changes, especially temperature fluctuations, can alter the composition of these bacterial communities, particularly in response to temperature fluctuations (Lima et al., 2020; Zhu et al., 2022; Ziegler et al., 2017). Our findings indicate that after the initial MHW, there was a significant increase in the abundance in the bacterial family u. Legionellaceae, with functional predictions suggesting that these include potentially pathogenic taxa and intracellular parasites. Legionellaceae are commonly found in coral reef ecosystems and have associations with coral pathogens (Vega et al., 2009), indicating that assumed beneficial bacterial groups within the coral symbiont community may transition into potentially pathogenic and intracellular parasites under high‐temperature conditions (McDevitt‐Irwin et al., 2017; Sweet et al., 2013). The density of coral‐associated symbiotic zooxanthellae and the content of Chl *a* were significantly reduced, while the levels of SOD, CAT, GST, and caspase‐3 were significantly increased in the coral tissues (Jiang et al., 2022). These findings indicate that HMWs can impact coral physiological functions and lead to an increase in the abundance of pathogenic bacteria, resulting in significant damage to the corals.

At the end of the first recovery phase, *Leptospira*, *Halomonas desiderata*, and u. Flavobacteriaceae maintained a relatively high abundance. The scientific literature suggests that *Leptospira* is a common intracellular parasite, frequently observed following extreme weather events (Cann et al., 2013; Kavela et al., 2023). Based on FAPROTAX predictions, it may have detrimental effects on corals (Vincent et al., 2019). *Halomonas desiderata* is an opportunistic microbe that forms a symbiotic relationship with corals, residing on the coral surface and utilizing organic matter present in the coral reef area, which helps corals resist invasion by pathogenic microorganisms (Wang & Shao, 2021). Marine heatwaves can lead to increased nitrogen and nitrate levels in coral reef areas (Grottoli et al., 2018; Howells et al., 2012; Wiedenmann et al., 2013). The symbiotic relationship between Flavobacteriaceae and corals is complex and sensitive (Certner & Vollmer, 2018; Meyer et al., 2016; Sweet et al., 2011; Vega et al., 2008). Based on functional predictions, u. Flavobacteriaceae is involved in organic matter decomposition. These results indicate that during the recovery period, although potentially pathogenic bacterial groups are present within the core community, beneficial bacterial groups rapidly increase to promote coral survival (Sun et al., 2023).

### 4.3 Bacteria adaptation during marine heatwaves

The microbiome flexibility hypothesis of metaorganism adaptation predicts that changes in microbial communities contribute to the response and adaptation to environmental fluctuations (Voolstra & Ziegler, 2020). In this study, following the second MHW, there was a significant increase in the abundance of certain bacteria.

There were increases in the abundances of u. *Coxiella*, u. Flavobacteriaceae, *Achromobacter arsenitoxydans*, and *Halomonas desiderata* under MHWs. *Coxiella* has been shown to cause diseases in invertebrates and is closely associated with coral health and disease (Antonio et al., 2000; Casas et al., 2004). u. Flavobacteriaceae was a core component of the community from the first to the second recovery phase, indicating its importance for coral health. Additionally, previous studies have demonstrated that *A. arsenitoxydans* and *H. desiderata* enhance the resistance of coral to environmental stress as stress‐tolerant bacteria (Abrego et al., 2008; Rosado et al., 2019). We found that, under the co‐regulation of coral and bacteria, the levels of SOD, CAT, GST, and caspase‐3 in coral tissues were reduced compared with those at the first MHW exposure (Yu et al., 2020).

These findings highlight the complex dynamics of the microbial community during the heatwave phases. Although repeated heatwaves can lead to the emergence of pathogenic bacteria, the abundance of beneficial bacterial taxa also increased. At the end of the second recovery phase, there were significant increases in the abundances of u. Dadabacteriales, u. Woeseia, *Rubritalea tangerine*, *uncultured delta proteobacterium*, and other beneficial microorganisms. These bacteria have important roles in coral health and growth (Jiang et al., 2019; Radecker et al., 2015; Roder, Arif, Bayer, et al., 2014; Roder, Arif, Daniels, et al., 2014; Röthig et al., 2016).

These strategic changes in the microbial community might contribute to adaptive responses to stress in coral, in which alterations in the microbial community improve survival in the MHW environment (Van‐Oppen & Blackall, 2019). This adaptability of the microbial community is crucial for supporting coral health and resilience in the face of stressful events. These findings have important implications in light of climate change as associated increases in MHWs.

## AUTHOR CONTRIBUTIONS

**Xin Zhai:** Formal analysis (equal); methodology (equal); software (equal); visualization (equal); writing – original draft (equal); writing – review and editing (equal). **YanPing Zhang:** Formal analysis (equal); funding acquisition (equal); methodology (equal); software (equal); validation (equal); visualization (equal); writing – review and editing (equal). **Jie Zhou:** Methodology (equal); software (equal); supervision (equal); validation (equal); writing – review and editing (equal). **Hao Li:** Formal analysis (equal); methodology (equal); software (equal); validation (equal). **Ao Wang:** Formal analysis (equal); software (equal); validation (equal); visualization (equal). **Li Liu:** Conceptualization (equal); funding acquisition (equal); methodology (equal); project administration (equal); supervision (equal); writing – review and editing (equal).

## ACKNOWLEDGEMENTS

We extend our heartfelt gratitude to all our collaborators who contributed their wisdom and efforts to this research, as well as to the staff of the editorial department and the reviewers who provided valuable advice and support. Their professional guidance and careful comments were essential to the refinement of our work and the ability to present it to you.

## FUNDING INFORMATION

This work was financially supported by the Guangdong Basic and Applied Basic Research Foundation (Grant No. A1515110225), Guangdong Innovation and Strengthening School Project (Grant No. 230419080), the Program for Scientific Research Start‐Up Funds of Guangdong Ocean University (Grant No. R18024), and Zhanjiang Science and Technology Planning Project (Grant No. B01005; 2021E05020), which we gratefully acknowledge, as well as the National Key R&D Project of China (Grant No. YFD2401302).

## CONFLICT OF INTEREST STATEMENT

The authors declare that they have no known competing financial interests or personal relationships that could have appeared to influence the work reported in this paper.

## Data Availability

The data that support the findings of this study are openly available in figshare: https://doi.org/10.6084/m9.figshare.24152901.
